# Evaluating dietary habits of adults and their relationship with sleep quality in the Kingdom of Saudi Arabia

**DOI:** 10.3389/fnut.2025.1664739

**Published:** 2025-10-27

**Authors:** Nouf A. Alghamdi, Arwa S. Almasaudi

**Affiliations:** ^1^Department of Community Health Sciences, College of Applied Medical Sciences, King Saud University, Riyadh, Saudi Arabia; ^2^Department of Clinical Nutrition, Faculty of Applied Medical Sciences, King Abdulaziz University, Jeddah, Saudi Arabia

**Keywords:** sleep quality, dietary habits, PSQI, sleep duration, sleep latency

## Abstract

**Introduction:**

Sleep plays a vital role in daily functioning and well-being, yet insufficient sleep is a growing global concern influenced by modern lifestyles.

**Methods:**

This study examined the relationship between dietary habits, and sleep quality among 1,041 Saudi adults using self-administered questionnaires, including the Pittsburgh Sleep Quality Index (PSQI) and a nutrition behavior questionnaire.

**Results:**

The key findings included that 77.4% of participants had poor sleep quality (PSQI > 6). Females reported worse sleep efficiency, more disturbances, and greater daytime dysfunction than males. Dietary patterns revealed low consumption of fruits (38.2%), vegetables (28%), fish (38.9%), and legumes (38%), and high consumption of starches (41%), poultry (26.4%), and sweets (29.9%). Positive associations were found between sleep efficiency and fruit, fish, and legume intake, while high starch, sweets, and dairy consumption correlated with poorer sleep quality, longer sleep latency, and increased daytime dysfunction. Gender-specific analysis showed distinct dietary effects. In males, fruits, vegetables, dairy, and legumes improved sleep quality, whereas starches and sweets negatively affected it. In females, sweets negatively affected sleep quality and latency, while fish consumption improved sleep efficiency and reduced dysfunction.

**Discussion:**

The study highlights the connection between diet and sleep, suggesting that individualized dietary interventions could help enhance sleep quality. However, limitations, such as self-reported data and confounding factors, call for further research using objective measures.

## 1 Introduction

Sleep is essential for daily functioning and overall well-being. It is characterized by reduced activity, closed eyes, and unconsciousness (1, 2). Insufficient sleep has become a growing global concern; over the past four decades, average sleep duration has declined by 2 h (3). This decrease is likely driven by lifestyle shifts, including increased activity levels and the widespread use of technology (4). Even brief periods of sleep deprivation can impair cognitive functions such as attention and working memory (4), likely due to hormonal changes that affect physical recovery and renewal (4, 5).

Common strategies to improve sleep include reducing light and noise and practicing relaxation techniques (5). While medications may aid sleep, they carry potential side effects and should be used cautiously (5). Tools such as the Pittsburgh Sleep Quality Index (PSQI) help assess sleep quality. The PSQI is a self-report questionnaire that measures sleep quality and disturbances over 1 month across seven components: subjective sleep quality, latency, duration, efficiency, disturbances, use of medication, and daytime dysfunction (6).

Research shows that poor sleep quality and short duration are linked to metabolic disorders (7–9) and unhealthy dietary patterns, including skipped meals and late-night snacking (10). Individuals with poor sleep often consume more food, exhibit irregular eating habits, and go to bed late (11, 12). One study found that those with poor sleep tended to skip breakfast and replace meals with snacks (13). Another reported increased energy and protein intake among long sleepers and higher saturated fat intake among short sleepers (13). Moreover, short sleep duration was associated with greater fat intake in men and higher carbohydrate intake in women, even after accounting for variables like age, activity level, smoking, and alcohol use (12).

In terms of sleep quality, diets high in protein (14), fiber-rich carbohydrates (15)— fish, and vegetables are associated with better sleep outcomes (16, 17). A study of middle-aged female Japanese workers found that low intake of vegetables and fish, high intake of sweets and noodles, and skipping breakfast were linked to poorer sleep (17). However, not all studies report consistent findings. For example, a cohort study from the Netherlands found no strong association between diet and sleep quality, suggesting cultural and population differences may play a role (18).

Despite the global evidence linking diet and sleep, few studies have examined this relationship in the Middle East, and particularly in Saudi Arabia. The Saudi population is undergoing rapid lifestyle transitions, including increased urbanization, dietary westernization, and high prevalence of sleep disturbances, making it a unique context to investigate. To our knowledge, no large-scale study in Saudi Arabia has comprehensively assessed the association between dietary habits and sleep quality, while also considering gender-specific differences. By addressing this gap, the present study contributes region-specific evidence that may inform culturally tailored dietary and public health strategies.

Understanding dietary habits, particularly macronutrient intake, is important for public health. Identifying nutritional imbalances enables tailored interventions, education, and policy. This study aimed to examine the association between diet and sleep quality among adults in Saudi Arabia, with a focus on gender-based differences.

## 2 Materials and methods

### 2.1 Study design

This cross-sectional study was approved by the Institutional Review Board (IRB) of King Saud University Medical City, Riyadh, Saudi Arabia (Reference no. E-22-6778). A convenience sample of 1,041 participants asked to complete a self-administered online questionnaire after providing informed consent.

### 2.2 Participants and recruitment

The inclusion criteria were being a male or female citizen or resident across various regions of Saudi Arabia aged ≥18 years or older. Data collection for the study occurred through an online questionnaire completed by participants between May and October 2023. The Microsoft Forms platform was used to collect data (Microsoft Corporation, Redmond, WA, United States), and the link was distributed through popular social media platforms, including WhatsApp (Facebook, Inc., Menlo Park, CA, United States), X (X, Inc., San Francisco, CA, United States), and LinkedIn (Mountain View, CA, United States).

To ensure broader regional representation, trained data collectors were assigned from the five major regions of Saudi Arabia (North, South, West, East, and Central). Each data collector was responsible for distributing the questionnaire link within their respective region. This strategy facilitated wider outreach across the Kingdom, minimized geographic clustering, and enhanced the diversity of responses while maintaining a convenience sampling design.

### 2.3 Questionnaire

The online questionnaire comprised three main sections to assess dietary behavior and sleep quality among adults in Saudi Arabia. It was administered in Arabic and took about 18 min to complete. Participants were first provided with an informed consent form outlining the study’s purpose, eligibility criteria, confidentiality measures, and estimated duration.

The first section gathered sociodemographic data, including age, gender, nationality, marital status, city of residence, employment status, education level, monthly income, living situation, chronic disease status, and smoking habits. Participants also self-reported their weight (kg) and height (cm), allowing for the calculation of body mass index (BMI).

The second section assessed sleep quality and patterns over the past month using the Arabic version of the PSQI (19). The questionnaire. This self-administered questionnaire included 19 items, five of which were answered by bedmates or roommates. It evaluates seven components: sleep quality, latency, duration, habitual efficiency, disturbances, use of sleep medication, and daytime dysfunction. Each component is scored from 0 to 3 (0 = no disturbance, 3 = severe disturbance). The total score (0–21) indicates global sleep quality, with scores above 5 suggesting poor sleep quality.

In section three, a validated, previously translated nutrition behavior questionnaire was used to assess participants’ dietary habits. It examined the consumption of various food groups, including starches, fruits, vegetables, dairy products, red meat, poultry, fish, and legumes. Participants reported consumption frequency using options from “I do not eat it at all” to “6 times/week.” They then reported typical portion sizes for each food group, choosing from: “I do not eat it at all,” “<1 portion,” “1 portion,” “2 portions,” “3–4 portions,” or “5 or more portions.” For each food group, participants were provided with examples of food items, along with the estimated size of one portion. For instance:

- Starch: ½ cup of cereal, 1 slice of bread, or 1 potato.
- Fruit: 1 whole fruit, ½ cup of juice, or dried fruit.
- Vegetables: 1 cup fresh or ½ cup cooked.
- Milk and dairy products: 1 glass or cup, or 1 slice of cheese.
- Red meat, poultry, and fish: 30 g.
- Legumes: 1 cup.

### 2.4 Statistical analysis

Data were analyzed using SPSS version 21.0 (IBM SPSS, Chicago, United States). Continuous variables were expressed as mean ± standard deviation, and categorical variables as frequencies and percentages. Chi-square and independent t-tests were used to assess differences between categorical and continuous variables, respectively. Spearman’s and Kendall’s tau-b correlations evaluated bivariate associations among PSQI scores, components, demographics, and dietary intake. A general linear model univariate analysis assessed global PSQI score differences across demographics, adjusted for covariates. Results were reported as coefficients (R). Figures were created in MS Excel. Statistical significance was set at *p* < 0.05.

## 3 Results

### 3.1 Characteristics of respondents

Table 1 shows the demographic characteristics of the respondents. Males were significantly older than females (*p* < 0.001), with most males aged 31–40 years (32.6%) and most females aged 21–30 years (41.8%). Males also had significantly higher BMIs and a greater prevalence of overweight and obesity (*p* < 0.001). Saudis made up 96% of the sample, with no significant gender difference. About 80% lived in shared accommodation, though males were more likely to live alone (29.7%) than females (15.1%; *p* < 0.001). While over half of all respondents were from the Central Region, most males came from the Western Region, and most females from the Central Region (*p* < 0.001). A total of 71.6% held a university degree; higher education was more common among males (21.5%) than females (6.8%; *p* < 0.001). Nearly 75% of males were employed compared to 27.7% of females (*p* < 0.001). Marriage was also more prevalent among males (60%) than females (36.9%; p < 0.001). Regarding income, 65% of females had no income or earned under 4,000 SAR/month versus 19.5% of males (*p* < 0.001). Both genders had poor mean PSQI scores, with females showing significantly worse sleep quality (*p* < 0.001).

**Table 1:** Demographic characteristics of respondents.

Parameters	All	Males	Females	*P*-value*
*N*	1,041	340	701	
Age (years)	31.1 ± 11.4	34.9 ± 10.5	29.3 ± 11.4	<0.001
BMI (kg/m^2^)	26.1 ± 6.0	27.2 ± 5.5	25.5 ± 6.1	<0.001
Age group (years)				
18–20	196 (18.8)	26 (7.6)	170 (24.3)	<0.001
21–30	398 (38.2)	105 (30.9)	293 (41.8)	
31–40	217 (20.8)	111 (32.6)	106 (15.1)	
41–50	154 (14.8)	68 (20.0)	86 (12.3)	
≥51	76 (7.3)	30 (8.8)	46 (6.6)	
BMI status				
Underweight	143 (13.7)	18 (5.3)	125 (17.8)	<0.001
Normal	345 (33.1)	104 (30.6)	241 (34.4)	
Overweight	315 (30.3)	127 (37.4)	188 (26.8)	
Obese class I	144 (13.8)	54 (15.9)	90 (12.8)	
Obese class II	94 (9.0)	37 (10.9)	57 (8.1)	
Nationality				
Saudis	1,000 (96.1)	329 (96.8)	671 (95.7)	0.42
Non-Saudis	41 (3.9)	11 (3.2)	30 (4.3)	
Residence				
Shared	834 (80.1)	239 (70.3)	595 (84.9)	<0.001
Alone	207 (19.9)	101 (29.7)	106 (15.1)	
Region				
East	47 (4.5)	14 (4.1)	33 (4.7)	<0.001
West	252 (24.2)	118 (34.7)	134 (19.1)	
North	135 (13.5)	84 (24.7)	51 (7.3)	
South	63 (6.1)	28 (8.2)	35 (5.0)	
Central	544 (52.3)	96 (28.2)	448 (63.9)	
Education				
Secondary or less	175 (16.8)	48 (14.1)	127 (18.1)	<0.001
University	745 (71.6)	219 (64.4)	526 (75.0)	
Post-Graduate	121 (11.6)	73 (21.5)	48 (6.8)	
Employment status				
Unemployed	82 (7.9)	16 (4.7)	66 (9.4)	<0.001
Housewife	91 (8.7)	--	91 (13.0)	
Student	359 (34.5)	52 (15.3)	307 (43.8)	
Freelance	23 (2.2)	11 (3.2)	12 (1.7)	
Employed	444 (42.7)	250 (73.5)	194 (27.7)	
Retired	42 (4.0)	11 (3.2)	31 (4.4)	
Marital status				
Single	548 (52.6)	131 (38.5)	417 (59.5)	<0.001
Married	463 (44.5)	204 (60.0)	259 (36.9)	
Divorced	26 (2.5)	5 (1.5)	21 (3.0)	
Widow/widower	4 (0.4)	0	4 (0.6)	
Monthly income (SAR)				
No income	175 (16.8)	20 (5.9)	155 (22.1)	<0.001
<2000	242 (23.2)	24 (7.1)	218 (31.1)	
2000–4,000	105 (10.1)	22 (6.5)	83 (11.8)	
4,001–7,000	109 (10.5)	55 (16.2)	54 (7.7)	
7,001–10,000	115 (11.0)	56 (16.5)	59 (8.4)	
>10,000	295 (28.3)	163 (47.9)	132 (18.8)	
Global PSQI Score	7.9 ± 3.2	7.3 ± 3.0	8.2 ± 3.2	<0.001

Data presented as frequencies (%) except age (mean ± SD); *denotes *p*-value for differences in males and females; *p*-value significant at <0.05.

### 3.2 Medical history characteristics of respondents

Table 2 presents the medical histories of the respondents. Multiple comorbidities were more common among females than males (*p* = 0.03). Dyslipidemia was the most frequent condition in both sexes (8.8% males, 9.1% females; *p* = 0.87). Among females, dyslipidemia was followed by depression (7.8%), anemia (7.7%), and gastrointestinal disorders (6.0%), all significantly higher than in males (4.1, 0.6, and 4.4%; *p* = 0.02, <0.001, and 0.29, respectively). Smoking prevalence was significantly higher in males (35.9%) than females (3.3%; *p* < 0.001). Hypertension was also more common in males (7.4%) than females (3.7%; *p* = 0.01). Anxiety was reported by nine participants, all females. Additionally, other unspecified diseases were more prevalent in females (*p* < 0.001).

**Table 2:** Medical history characteristics of respondents.

Parameters	All	Males	Females	*P*-value
*N*	1,041	340	701	
No. of comorbidities				
None	664 (63.8)	237 (69.7)	427 (60.9)	0.03
1	252 (24.2)	75 (22.1)	177 (25.2)	
2	86 (8.3)	18 (5.3)	68 (9.7)	
3	31 (3.0)	9 (2.6)	22 (3.1)	
≥4	8 (0.8)	1 (0.3)	7 (1.0)	
Diabetes Mellitus	47 (4.5)	18 (5.3)	29 (4.1)	0.40
Depression	69 (6.6)	14 (4.1)	55 (7.8)	0.02
Anemia	56 (5.4)	2 (0.6)	54 (7.7)	<0.001
Dyslipidemia	94 (9.0)	30 (8.8)	64 (9.1)	0.87
Hypertension	51 (4.9)	25 (7.4)	26 (3.7)	0.01
Respiratory Diseases	46 (4.4)	14 (4.1)	32 (4.6)	0.74
Heart Disease	10 (1.0)	3 (0.9)	7 (1.0)	0.86
Gastrointestinal diseases	57 (5.5)	15 (4.4)	42.0 (6.0)	0.29
Liver Disease	6 (0.6)	2 (0.6)	4 (0.6)	0.97
Hypothyroidism	12 (1.2)	0	12 (1.7)	0.01
Kidney Disease	7 (0.7)	4 (1.2)	3 (0.4)	0.17
Anxiety	9 (0.9)	0	9 (1.3)	0.04
Smoker	145 (13.9)	122 (35.9)	23 (3.3)	<0.001
Other diseases	64 (6.1)	6 (1.8)	58 (8.3)	<0.001

Data presented as frequencies (%); *p*-value significant at <0.05.

### 3.3 PSQI components

**Figure 1:**
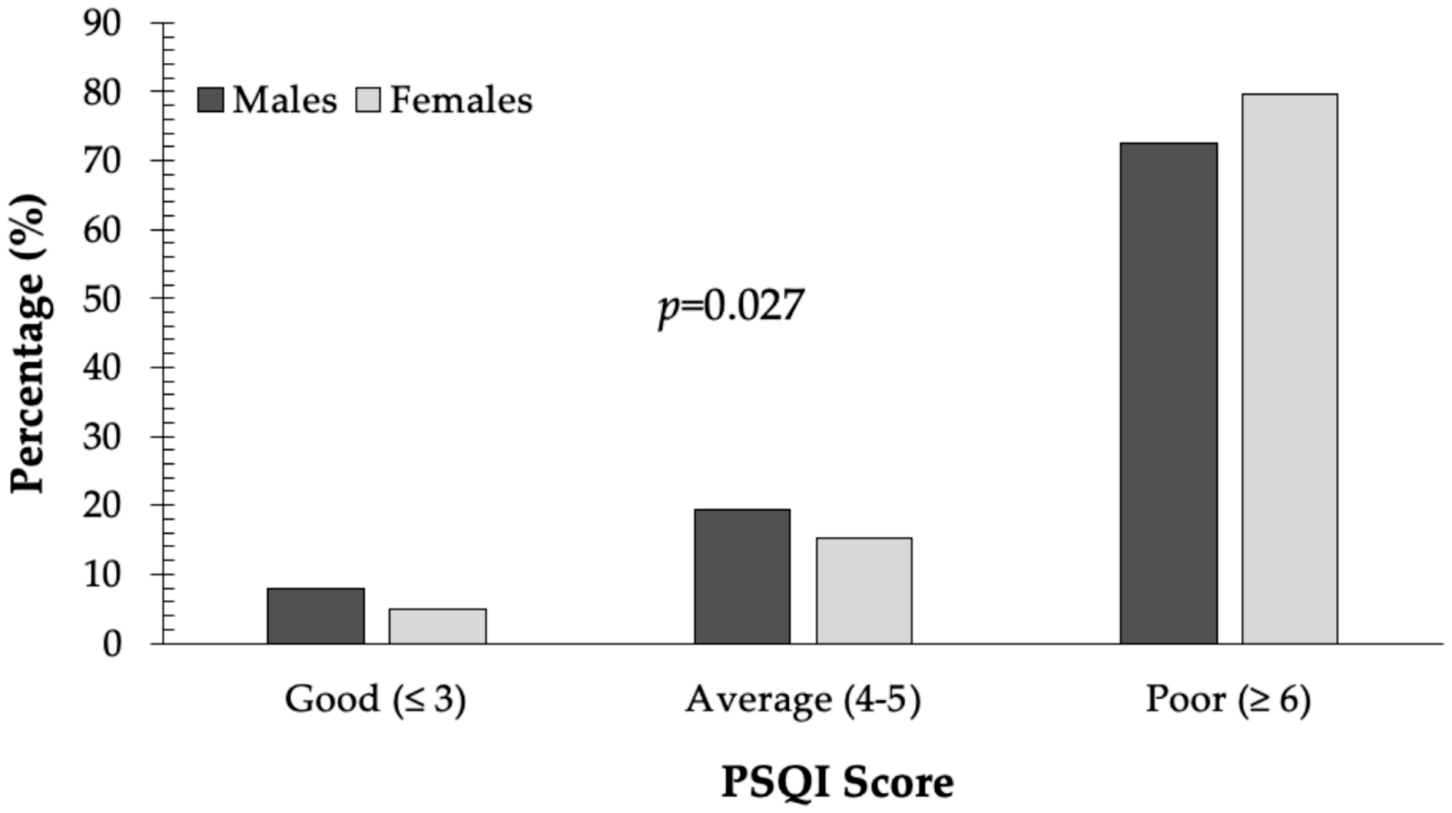
PSQI status in males and females.

An overwhelming 77.4% of respondents had PSQI scores above 6, while only 6% were categorized as having “good” sleep quality. Poor sleep was significantly more prevalent in females than males (79.7% vs. 72.6%; *p* = 0.027) (Figure 1).

Table 3 presents sleep characteristics based on the seven PSQI components. Only 26.4% reported very good subjective sleep quality, with males slightly better than females (31.2% vs. 24.1%; *p* = 0.08). About 12% reported very poor sleep quality. No gender differences were found in sleep latency, though 17.8% had severe latency. Over 75% slept 6+ h, but nearly half of males reported 6–7 h of sleep, compared to 36% of females (*p* = 0.007). Females had significantly higher rates of <65% sleep efficiency (51.8% vs. 45.6%; *p* = 0.01), moderate to severe sleep disturbances (35.6% vs. 26.9%; *p* < 0.001), and daytime dysfunction (31.1% vs. 19.4%; *p* < 0.001). Medication use was similar between genders, with over 80% reporting no use in the past month (Table 3).

**Table 3:** PSQI components.

Component	All	Males	Females	*P*-value
Subjective sleep quality				
Very good	275 (26.4)	106 (31.2)	169 (24.1)	0.08
Fairly good	517 (49.7)	156 (45.9)	361 (51.5)	
Fairly poor	122 (11.7)	35 (10.3)	87 (12.4)	
Very poor	127 (12.2)	43 (12.6)	84 (12.0)	
Sleep latency				
Low	202 (19.4)	63 (18.5)	139 (19.8)	0.58
Mild	356 (34.2)	126 (37.1)	230 (32.8)	
Moderate	298 (28.6)	95 (27.9)	203 (29.0)	
Severe	185 (17.8)	56 (16.5)	129 (18.4)	
Sleep duration				
>7 h	382 (36.7)	109 (32.1)	273 (38.9)	0.007
6–7 h	412 (39.6)	160 (47.1)	252 (35.9)	
5–6 h	149 (14.3)	41 (12.1)	108 (15.4)	
<5 h	98 (9.4)	30 (8.8)	68 (9.7)	
Sleep efficiency				
>85%	315 (30.3)	125 (36.8)	190 (27.1)	0.01
75–84%	159 (15.3)	49 (14.4)	110 (15.7)	
65–74%	49 (4.7)	11 (3.2)	38 (5.4)	
<65%	518 (49.8)	155 (45.6)	363 (51.8)	
Sleep disturbances				
Low	76 (7.3)	37 (10.9)	39 (5.6)	0.001
Mild	628 (60.3)	215 (63.2)	413 (58.9)	
Moderate	298 (28.6)	81 (23.8)	217 (31.0)	
Severe	39 (3.7)	7 (2.1)	32 (4.6)	
Use of sleep medications				
Not during past month	867 (83.3)	279 (82.1)	588 (83.9)	0.80
Less than once a week	100 (9.6)	33 (9.7)	67 (9.6)	
Less than twice last week	42 (4.0)	16 (4.7)	26 (3.7)	
Three or more times a week	32 (3.1)	12 (3.5)	20 (2.9)	
Daytime dysfunction				
Low	235 (22.6)	106 (31.2)	129 (18.4)	<0.001
Mild	522 (50.1)	168 (49.4)	354 (50.5)	
Moderate	221 (21.2)	57 (16.8)	164 (23.4)	
Severe	63 (6.1)	9 (2.6)	54 (7.7)	

Data presented as frequencies (%); significance at p < 0.05.

**Figure 2:**
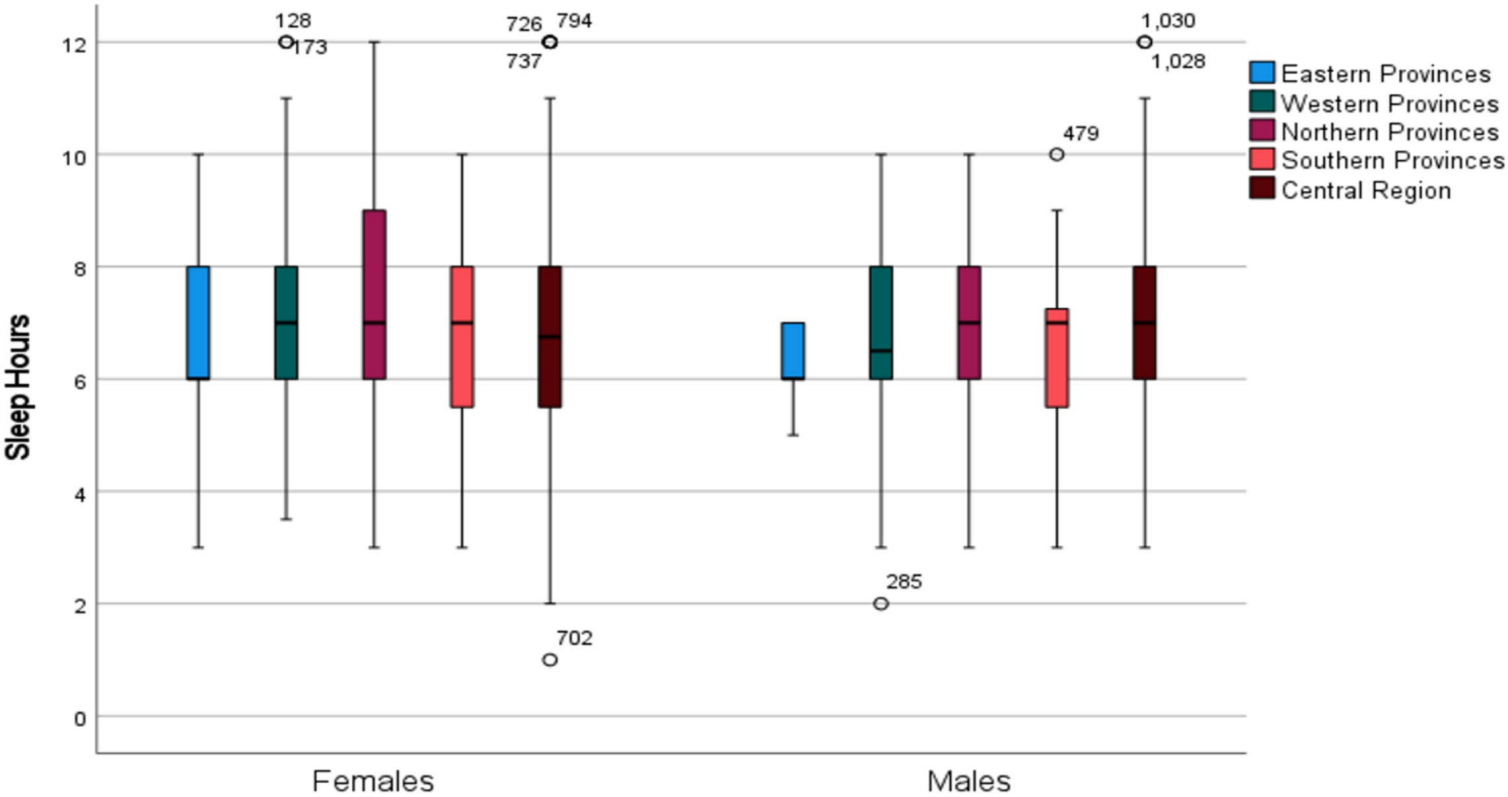
Over-all mean global PSQI Hours according to region in males and females.

Interestingly, males from the Eastern Region reported the longest sleep durations compared to those from other regions, even after adjusting for age and BMI (Figure 2), a pattern not observed in females (*p* = 0.17).

### 3.4 Dietary intake of respondents

Table 4 presents weekly consumption frequencies of major food types. Most respondents consumed less than one portion per week of fruits (38.2%), vegetables (28%), fish (38.9%), and legumes (38%). Frequently consumed foods (≥7 portions/week) included starches (41%), poultry (26.4%), and sweets (29.9%). Significant gender differences emerged: males consumed fewer fruits, vegetables, and dairy, while females more frequently consumed starches, poultry, and sweets (Table 4).

**Table 4:** Food intake of respondents.

Food type	Frequency of intake					
	I do not eat at all	<1 portion per week	1–2 portions per week	3–4 portions per week	5–6 portions per week	≥7 portions per week
All respondents						
Fruits	125 (12.0)	398 (38.2)	270 (25.9)	116 (11.1)	45 (4.3)	87 (8.4)
Vegetables	82 (7.9)	291 (28.0)	270 (25.9)	160 (15.4)	92 (8.8)	146 (14.0)
Dairy and milk products	91 (8.7)	211 (20.3)	255 (24.5)	150 (14.4)	105 (10.1)	229 (22.0)
Starches	27 (2.6)	118 (11.3)	183 (17.6)	139 (13.4)	147 (14.1)	427 (41.0)
Red meat	113 (10.9)	250 (24.0)	262 (25.2)	163 (15.7)	94 (9.0)	159 (15.3)
Poultry	51 (4.9)	149 (14.3)	212 (20.4)	212 (20.4)	142 (13.6)	275 (26.4)
Fish	282 (27.1)	405 (38.9)	194 (18.6)	60 (5.8)	48 (4.6)	52 (5.0)
Legumes	178 (17.1)	396 (38.0)	253 (24.3)	93 (8.9)	57 (5.5)	64 (6.1)
Sweets and candies	77 (7.4)	185 (17.8)	223 (21.4)	146 (14.0)	99 (9.5)	311 (29.9)
Males						
Fruits	43 (12.6) *	135 (39.7)	103 (30.3)	28 (8.2)	12 (3.5)	19 (5.6)
Vegetables	37 (10.9) *	104 (30.6)	92 (27.1)	40 (11.8)	30 (8.8)	37 (10.9)
Dairy and milk products	43 (12.6) **	77 (22.6)	77 (22.6)	59 (17.4)	31 (9.1)	53 (15.6)
Starches	16 (4.7) **	50 (14.7)	58 (17.1)	54 (15.9)	41 (12.1)	121 (35.6)
Red meat	22 (6.5) *	82 (24.1)	86 (25.3)	64 (18.8)	31 (9.1)	55 (16.2)
Poultry	13 (3.8)	54 (15.9)	62 (18.2)	73 (21.5)	56 (16.5)	82 (24.1)
Fish	84 (24.7)	138 (40.6)	63 (18.5)	22 (6.5)	15 (4.4)	18 (5.3)
Legumes	57 (16.8)	121 (35.6)	83 (24.4)	39 (11.5)	22 (6.5)	18 (5.3)
Sweets and candies	37 (10.9) **	86 (25.3)	68 (20.0)	44 (12.9)	25 (7.4)	80 (23.5)
Females						
Fruits	82 (11.7)	263 (37.5)	167 (23.8)	88 (12.6)	33 (4.7)	68 (9.7)
Vegetables	45 (6.4)	187 (26.7)	178 (25.4)	120 (17.1)	62 (8.8)	109 (15.5)
Dairy and milk products	48 (6.8)	134 (19.1)	178 (25.4)	91 (13.0)	74 (10.6)	176 (25.1)
Starches	11 (1.6)	68 (9.7)	125 (17.8)	85 (12.1)	106 (15.1)	306 (43.7)
Red meat	91 (13.0)	168 (24.0)	176 (25.1)	99 (14.1)	63 (9.0)	104 (14.8)
Poultry	38 (5.4)	95 (13.6)	150 (21.4)	139 (19.8)	86 (12.3)	193 (27.5)
Fish	198 (28.2)	267 (38.1)	131 (18.7)	38 (5.4)	33 (4.7)	34 (4.9)
Legumes	121 (17.3)	275 (39.2)	170 (24.3)	54 (7.7)	35 (5.0)	46 (6.6)
Sweets and candies	40 (5.7)	99 (14.1)	155 (22.1)	102 (14.6)	74 (10.6)	231 (33.0)

Data presented as frequencies (%); * denotes significance compared to females at 0.05 level and ** at 0.01 level. Actual *p*-values are 0.02 for fruits, 0.01 for vegetables, <0.001 for dairy and milk products; 0.001 for starches; 0.03 for red meat; 0.19 for poultry 0.85 for fish; 0.31 for legumes and <0.001 for sweets and candies.

### 3.5 Associations of PSQI components according to dietary habits

Kendall’s tau-b rank correlations revealed significant associations between dietary intake and PSQI score status among all respondents (Table 5). Specifically, starch intake (*r* = 0.06; *p* < 0.05) and sweets and candies (*r* = 0.08; *p* < 0.01) were positively associated with higher PSQI scores, indicating poorer sleep. When stratified by gender, these associations remained significant only in males, with red meat also significantly correlated with PSQI scores (*r* = 0.11; *p* < 0.05). No significant associations were found in females.

**Table 5:** Associations of PSQI components according to eating habits.

Food intake	Global PSQI score	PSQI components						
		Subjective sleep quality	Sleep latency	Sleep duration	Sleep efficiency	Sleep disturbance	Use of sleep medications	Daytime dysfunction
All								
Fruits	0.03	−0.05	0.007	0.02	0.10**	−0.004	−0.02	−0.03
Vegetables	0.01	0.002	0.05	−0.002	0.05	0.02	0.001	−0.02
Dairy and milk products	0.03	0.02	0.001	−0.03	−0.02	0.001	−0.002	0.10**
Starches	0.06*	0.06	0.04	−0.03	−0.008	0.009	−0.05	0.17**
Red meat	0.04	−0.05	−0.02	−0.04	0.04	−0.05	0.001	−0.04
Poultry	0.03	0.004	0.02	−0.009	0.02	−0.005	−0.05	0.05
Fish	0.01	−0.01	0.03	−0.004	0.07*	0.007	0.09**	−0.08
Legumes	0.04	0.01	0.002	0.02	0.11**	0.007	0.05	−0.04
Sweets and candies	0.08**	0.07*	0.09**	−0.003	−0.03	0.05	−0.04	0.16**
Males								
Fruits	0.003	−0.15**	−0.11*	−0.04	0.12**	−0.06	0.09	−0.07
Vegetables	−0.005	−0.14**	−0.11*	−0.08	0.12**	−0.06	0.07	−0.05
Dairy and milk products	0.03	−0.11*	−0.10*	−0.08	0.006	0.04	−0.06	−0.03
Starches	0.11*	−0.001	0.02	−0.003	0.01	0.12**	−0.01	0.06
Red Meat	0.11*	−0.05	0.05	0.05	0.07	0.08	0.04	0.03
Poultry	0.05	−0.08	0.05	−0.02	0.05	0.09	−0.03	0.02
Fish	0.03	−0.05	0.007	0.02	0.02	0.04	0.08	−0.09
Legumes	0.02	−0.11*	−0.08	−0.04	0.07	0.03	−0.01	−0.05
Sweets and candies	0.09*	0.06	0.14**	0.06	−0.09	0.15**	−0.08	0.03
Females								
Fruits	0.04	−0.05	0.007	0.02	0.10**	−0.004	−0.02	−0.03
Vegetables	0.01	0.002	0.04	−0.001	0.05	0.02	−0.002	−0.02
Dairy and milk products	0.01	0.01	−0.003	−0.04	0.02	−0.001	0.01	0.11**
Starches	0.02	0.04	0.04	−0.03	−0.005	0.005	−0.06	0.16**
Red meat	0.01	−0.05	−0.02	−0.05	0.04	−0.05	−0.001	−0.04
Poultry	0.02	−0.005	0.01	−0.01	0.02	−0.005	−0.04	0.05
Fish	0.01	−0.01	−0.03	−0.004	0.08*	0.007	0.09**	−0.08**
Legumes	0.05	0.01	0.002	0.02	0.11**	0.007	0.05	−0.04
Sweets and candies	0.06	0.07*	0.10**	0.005	−0.03	0.04	−0.04	0.16**

Data presented as coefficient (R); * denotes significance at 0.05 level; ** denotes significance at 0.01 level.

Analysis using PSQI components (Table 5) showed that, among all participants, fruit intake was significantly associated with sleep efficiency (*r* = 0.10; *p* < 0.01), as were dairy products (*r* = 0.10; *p* < 0.01), starches (*r* = 0.17; *p* < 0.01), sweets and candies (*r* = 0.16; *p* < 0.01), and daytime dysfunction. Fish intake was associated with better sleep efficiency (*r* = 0.07; *p* < 0.05) and greater use of sleep medications (*r* = 0.09; *p* < 0.01), while legumes were significantly associated with sleep efficiency (*r* = 0.11; *p* < 0.01). Sweets and candies were the only item linked to subjective sleep quality (*r* = 0.07; *p* < 0.05) and sleep latency (*r* = 0.09; *p* < 0.01). Vegetables, red meat, and poultry were not associated with any PSQI components.

Table 5 shows gender-specific correlations. In males, higher intake of fruits, vegetables, dairy, and legumes was significantly and inversely associated with subjective sleep quality. Fruits, vegetables, and dairy were also inversely related to sleep latency. Fruit and vegetable intake was significantly associated with better sleep efficiency. Conversely, higher intake of sweets and candies was significantly associated with longer sleep latency, and both sweets and starches were linked to more sleep disturbances (all *p*-values < 0.05).

In females, Table 5 shows higher intake of sweets and candies was associated with worse subjective sleep quality and sleep latency. Better sleep efficiency was significantly linked to intake of fruits, fish, and legumes. Fish intake was also related to greater use of sleep medications. Daytime dysfunction was associated with higher intake of dairy, starches, and sweets, and inversely with fish intake (all *p*-values < 0.05).

## 4 Discussion

This study examined the relationship between dietary patterns and sleep quality among Saudi adults, focusing on food group consumption frequency and its association with various sleep quality components. Gender-specific analyses were also conducted. Notably, 77.4% of participants had PSQI scores above 6, indicating poor sleep, while only 6% reported good sleep quality. This finding raises public health concerns, as poor sleep is linked to chronic conditions like cardiovascular disease and metabolic syndrome. For instance, meta-analyses suggest that both short and long sleep durations may increase obesity, hypertension, and hyperglycemia risks, emphasizing sleep as a critical, modifiable factor in metabolic health (20, 21).

When stratifying data by gender, we found that poor PSQI scores were significantly more prevalent in females than males. Women also scored worse on specific PSQI components, including sleep efficiency, sleep disturbances, and daytime dysfunction. Females were more likely to report lying awake for over 35% of the night and experiencing moderate to severe disruptions and impaired daytime performance due to poor sleep. These findings suggest that women in this population experience poorer overall sleep quality and functioning.

These results align with previous studies reporting gender differences in sleep. A population-based study in Hunan Province, China, found that women had higher PSQI component scores than men, indicating worse sleep quality (22). Another study similarly showed that women reported poorer sleep than men (23). These differences may be partly explained by biological and hormonal factors, such as fluctuations in estrogen and progesterone throughout the menstrual cycle, pregnancy, and menopause. Hormonal shifts can influence sleep quality, sleep latency, and sleep architecture. For example, declining estrogen levels during menopause are associated with sleep disturbances, including insomnia and night sweats. Hormonal fluctuations can also reduce rapid eye movement (REM) sleep and prolong sleep onset time, contributing to reduced overall sleep quality (24).

Beyond biological and hormonal factors, gender differences in stress response and environmental influences may also contribute to disparities in sleep patterns. Interestingly, medication use was low among both sexes, with over 80% not regularly using sleep aids, suggesting that pharmacological interventions are unlikely to explain the poor sleep quality observed.

Our findings also revealed a low intake frequency of health-promoting food groups. A substantial proportion of participants consumed fruits (38.2%), vegetables (28%), fish (38.9%), and legumes (38%) less than once per week. In contrast, the most frequently consumed food types, defined as seven or more servings per week, included starches (41%), poultry (26.4%), and sweets (29.9%). These trends are concerning, as a higher intake of nutrient-rich foods is linked to improved sleep and overall health outcomes (25). The observed dietary pattern suggests preferences that may be shaped by availability, cultural norms, and nutritional awareness.

Gender-based differences in dietary habits were also evident. Males consumed fewer fruits, vegetables, and dairy products than females. This is consistent with previous research showing that men tend to consume more total calories but favor energy-dense foods while eating fewer fruits and vegetables, reflecting differing eating styles and health behaviors (26, 27).

In this study, females reported more frequent consumption of starches, poultry, and sweets compared to males, a pattern that may reflect social and cultural influences on dietary habits. Previous studies suggest women are more likely to choose foods perceived as healthy and often take a leading role in household food choices (28). Their higher intake of fruits and vegetables and lower fat consumption has been linked to motivations around health and weight control, whereas men tend to prefer meat and energy-dense foods (26, 27). These gender-specific dietary behaviors influence nutritional status and health outcomes, with men’s higher intake of calorie-dense, nutrient-poor foods potentially contributing to elevated obesity and disease risk (26).

Diet plays a role in multiple sleep parameters. In this study, fruit intake was significantly associated with improved sleep efficiency. This aligns with research showing that fruits rich in antioxidants and melatonin precursors, such as tryptophan and serotonin, can enhance sleep quality (10). Kiwi fruit, for example, improves sleep duration and efficiency due to its serotonin content (29). Additionally, fish intake was associated with better sleep efficiency and greater sleep medication use, likely due to omega-3 and vitamin D’s role in melatonin regulation (30). Legumes, rich in magnesium and fiber, were also positively associated with sleep efficiency (31).

Milk and other dairy products, along with starches, sweets, and candies, were significantly associated with increased daytime dysfunction. High intake of sugars and starches can lead to postprandial blood sugar crashes, contributing to daytime fatigue and reduced cognitive performance (32). Dairy products contain tryptophan, which may improve sleep when consumed moderately, but their effect could be diminished or even negative when combined with high-calorie or sugary foods. Sweets and candies were uniquely linked to subjective sleep quality and sleep latency (33). Excessive sugar consumption may disrupt sleep onset and quality by causing blood sugar fluctuations and promoting inflammation, both detrimental to sleep (34).

Interestingly, intake of vegetables, red meat, and poultry showed no significant associations with any PSQI components. Although vegetables are generally health-promoting, their specific impact on sleep may vary based on type (e.g., leafy greens vs. starchy vegetables) and preparation methods (35). Similarly, the lack of associations for red meat and poultry may reflect complex dietary-metabolic interactions (36). Nonetheless, recent studies have reported significant links between vegetable intake and sleep duration (37), as well as red meat consumption and sleep quality (38, 39).

The relationship between dietary patterns and sleep quality varied notably between males and females, emphasizing the importance of sex-specific dietary influences on sleep. Across all respondents, higher PSQI scores (indicating poorer sleep quality) were significantly associated with the intake of starches, sweets, and candies. These findings align with previous research suggesting that diets high in refined carbohydrates and sugars disrupt sleep due to fluctuations in blood glucose and hormonal imbalances (10). However, gender stratification revealed notable differences, highlighting the complex interplay between diet, biological sex, and sleep.

In males, starch, sweets, candies, and red meat intake were significantly associated with PSQI scores. These results align with studies linking red meat consumption to poorer sleep quality, possibly due to saturated fat and energy density, which may affect sleep architecture. Intake of fruits, vegetables, dairy products, and legumes showed an inverse relationship with subjective sleep quality, with higher consumption linked to better sleep and shorter sleep latency. This likely reflects the vitamins, antioxidants, and tryptophan content that support serotonin and melatonin synthesis (40, 41). Fruits and vegetables were also associated with improved sleep efficiency. Conversely, sweets and candies correlated with increased sleep latency, delaying sleep onset. Additionally, starches and sweets were linked to sleep disturbances, supporting evidence that high-glycemic foods may cause nighttime awakenings (42).

In females, the consumption of sweets and candies significantly were associated with both subjective sleep quality and sleep latency. This suggests that higher intake of these foods worsens sleep parameters, consistent with evidence that high sugar consumption disrupts sleep continuity and increases nighttime arousal (43). Daytime dysfunction was significantly associated with higher intake of dairy products (44), starches (45), and sweets, whereas fish consumption showed an inverse association (17). Fish, rich in omega-3 fatty acids, is linked to improved sleep regulation due to its effects on melatonin secretion (46, 47).

These findings highlight the crucial role of dietary habits in sleep quality among Saudi adults and emphasize the need for targeted interventions. Public health programs should prioritize gender-specific strategies: encouraging men to increase consumption of fruits, vegetables, and legumes while reducing starches and sweets, and advising women to limit sweets and increase fish intake to enhance sleep efficiency. Clinicians should integrate dietary assessments into sleep evaluations, providing personalized recommendations based on nutritional patterns. Such dietary adjustments can be part of broader approaches to managing sleep disorders and improving overall health. Policymakers can support these initiatives through clearer food labeling and subsidies for nutrient-rich foods, such as fish and legumes, aligning with broader health goals under Saudi Vision 2030.

This study has limitations. First, reliance on self-administered online questionnaires introduces potential reporting bias. Participants might misreport dietary habits, sleep quality, weight, or height due to social desirability, recall errors, or misunderstanding questions. Although the PSQI is validated, some items require bedmate or roommate responses, which may not have been feasible for all. Portion size estimates in the nutrition questionnaire may not accurately reflect actual intake, as participants’ perceptions vary despite examples.

Another limitation is that no statistical correction was applied for multiple comparisons. Given the exploratory nature of this study, we aimed to identify potential associations; however, this increases the risk of type I error, and findings should be interpreted with caution. In addition, as this was a cross-sectional study, the observed associations cannot establish causality, and longitudinal or interventional studies are needed to confirm these relationships. An important limitation of this study is that potential confounding variables, including physical activity levels and psychological health, were not measured. Although we statistically adjusted for age, sex, BMI, and smoking, residual confounding may persist due to these unaccounted factors. For example, stress or sedentary behavior could independently influence both dietary habits and sleep quality. Future studies should incorporate these variables to strengthen causal inference.

Although our regionally stratified sampling enhanced geographic diversity, the social media-based convenience approach limits generalizability, particularly for rural populations and older adults with limited digital access.

In addition, emerging evidence suggests BMI and vitamin D status may mediate the diet-sleep relationship, particularly in Saudi Arabia, where vitamin D deficiency and obesity rates are high. These factors could interact with diet to affect sleep via metabolic, hormonal, and circadian pathways. Future studies should explore these mechanisms. Recent work by Gong et al. supports the importance of considering such pathways to advance innovation in this field (48). These limitations call for cautious interpretation and suggest areas for future research.

However, the study has several strengths, including a large sample size of 1,041 participants, which improves the reliability and generalizability of the findings. The use of validated tools, such as the PSQI and a nutrition behavior questionnaire, adds credibility since these are established methods for assessing sleep quality and dietary habits. The detailed sociodemographic data allows for an in-depth evaluation of various influencing factors. Additionally, the gender-specific analysis offers insights into how dietary impacts on sleep quality differ between males and females, enabling tailored recommendations.

## 5 Conclusion

This study highlights a significant link between dietary patterns and sleep quality in adults, with clear gender differences. It emphasizes the potential of targeted dietary interventions to improve sleep, especially for higher-risk groups like females. However, some food groups, such as vegetables, red meat, and poultry, showed no significant impact, indicating that not all foods affect sleep equally. Men and women respond differently to the same foods, demonstrating gender-specific effects of diet on sleep. These findings underscore the complex relationship between nutrition and sleep and call for further research to guide evidence-based dietary recommendations for better sleep and health.

## Data Availability

The original contributions presented in the study are included in the article/supplementary material, further inquiries can be directed to the corresponding author.
